# Value of surgical pilot and feasibility study protocols

**DOI:** 10.1002/bjs.11167

**Published:** 2019-05-10

**Authors:** K. Fairhurst, J. M. Blazeby, S. Potter, C. Gamble, C. Rowlands, K. N. L. Avery

**Affiliations:** ^1^ Centre for Surgical Research and Medical Research Council (MRC) ConDuCT‐II Hub for Trials Methodology Research, Department of Population Health Sciences, Bristol Medical School University of Bristol Bristol UK; ^2^ MRC North West Hub for Trials Methodology Research University of Liverpool Liverpool UK

## Abstract

**Background:**

RCTs in surgery are challenging owing to well established methodological issues. Well designed pilot and feasibility studies (PFS) may help overcome such issues to inform successful main trial design and conduct. This study aimed to analyse protocols of UK‐funded studies to explore current use of PFS in surgery and identify areas for practice improvement.

**Methods:**

PFS of surgical interventions funded by UK National Institute for Health Research programmes from 2005 to 2015 were identified, and original study protocols and associated publications sourced. Data extracted included study design characteristics, reasons for performing the work including perceived uncertainties around conducting a definitive main trial, and whether the studies had been published.

**Results:**

Thirty‐five surgical studies were identified, of which 29 were randomized, and over half (15 of 29) included additional methodological components (such as qualitative work examining recruitment, and participant surveys studying current interventions). Most studies focused on uncertainties around recruitment (32 of 35), with far fewer tackling uncertainties specific to surgery, such as intervention stability, implementation or delivery (10 of 35). Only half (19 of 35) had made their results available publicly, to date.

**Conclusion:**

The full potential of pretrial work to inform and optimize definitive surgical studies is not being realized.

## Introduction

High‐quality RCTs are necessary to inform evidence‐based surgical practice. Surgical trials are challenging to do owing to established methodological issues extending beyond those of RCTs in other areas^1^, ^2^, ^3^, ^4^. Challenges specific to surgical RCTs include uncertainties around the stability and/or standardization of the intervention, selection and/or measurement of relevant clinical and patient‐reported outcomes, and issues surrounding patient recruitment such as clinician and patient equipoise^5^, ^6^, ^7^. These specific challenges, combined with surgeons' lack of familiarity with RCTs and the perception that participation can be onerous, may mean that trials are not initiated, conducted efficiently or completed on time and on target.

Well designed pilot and feasibility studies (PFS) may help to overcome challenges associated with undertaking RCTs in surgery, by allowing uncertainties to be addressed, and optimal design of the main trial to be determined^8^, ^9^, ^10^. Previous methodological work has considered the use and misuse of PFS in general. Several checklists have been developed^11^, ^12^, ^13^, ^14^, ^15^, ^16^ to identify and categorize specific reasons for undertaking pilot work, with the aim of guiding researchers into considering these for their study. More recently, guidelines for improved reporting of PFS, in the form of an extension to the CONSORT statement^17^, have been published. Integral to the development of these guidelines was the publication of a conceptual framework^18^ to promote understanding by defining the purpose and scope of different types of PFS in preparation for RCTs. These guidelines were, however, mostly theoretical, with no specific recommendations for the use of PFS in surgery.

Some published recommendations have provided more practical guidance regarding the use of PFS in surgery. The Medical Research Council (MRC) framework^19^ for developing and evaluating all complex interventions (defined as interventions with multiple components acting both independently and interdependently) includes surgery. Within this framework, undertaking PFS before full‐scale evaluation of surgical interventions in a definitive trial is considered vital preparatory work^19^. The IDEAL (Idea, Development, Exploration, Assessment and Long‐term follow‐up) framework^20^, ^21^, ^22^ also provides recommendations specific to the evaluation of novel surgical interventions from first in man to long‐term studies, and suggests study designs and issues to be considered at each stage of evaluation. Both the MRC and, more specifically for surgery, the IDEAL recommendations discuss PFS as part of a larger framework for the development and assessment of new complex interventions, and list several elements of the design of PFS.

Although PFS are thought to be beneficial and are endorsed as part of the strategic guidance discussed, there is uncertainty regarding exactly how they influence the design and conduct of successful RCTs^22^. At a fundamental level, published literature suggests that the wider surgical community may not understand the concept of PFS, with evidence of small, underpowered RCTs often mislabelled as pilot or feasibility studies^11^, ^14^, ^23^. Such studies often fail to address baseline feasibility issues such as considering whether a main trial is possible, and instead focus on formal hypothesis testing^24^, ^25^, ^26^, ^27^, ^28^. Further work is therefore needed to understand when and how PFS may be used optimally to inform future main trials in surgery.

Development of guidance that addresses the challenges associated with undertaking surgical PFS requires understanding of the current use of such studies in surgery. As PFS are often poorly reported, a traditional systematic review of surgical PFS is unlikely to be informative beyond what is already known. Major research funders are increasingly recognizing the importance of well designed PFS in informing main trial design. It was therefore hypothesized that protocols of competitively funded PFS may provide more informative insights into the current use of PFS in surgery, and into how pretrial work may be used to inform future definitive studies. The aim of this paper was to analyse protocols of successfully funded surgical PFS. The purpose of this review was to identify how PFS are currently used, consider whether their use is appropriate, and envisage how the use of PFS could be further improved.

## Methods

A systematic analysis of the protocols of PFS of surgical interventions funded by the UK National Institute for Health Research (NIHR) was undertaken.

### Characteristics of information sources, search strategy and screening

The UK NIHR Health Technology Assessment (HTA) and Research for Patient Benefit (RfPB) programmes were selected to identify PFS of surgical interventions. These programmes are established major national funders of high‐quality patient‐centred research, having funded trials for 25 and 12 years respectively. Both programmes fund definitive evaluations of the clinical and cost‐effectiveness of interventions, as well as feasibility studies to inform future definitive trials. They have publicly available and searchable databases of funded studies^29^, ^30^. Given the scope and longevity of both programmes, it was hypothesized that each would have funded surgical PFS, providing a sample of potentially well designed work from which to explore the role of PFS in surgery, and study their impact on main trial design and conduct.

The HTA and RfPB databases were searched for surgical PFS. Titles and abstracts were screened in duplicate, with any issues resolved by discussion and/or with senior input where necessary. Agreement was reached on inclusion or exclusion for 1283 studies (95·7 per cent) at the first attempt. Protocols for all included HTA studies were downloaded from the HTA website and those for all included RfPB studies (apart from 1 available online) were obtained by contacting the chief investigator of each study directly. Additional publications relating to included studies were identified by searching for links to published outputs on the NIHR website (HTA only), and using the study title, acronym and chief investigator name to search on PubMed, Google Scholar and the ISRCTN trials registry online.

### Inclusion and exclusion criteria

Protocols of all surgical PFS funded by the NIHR HTA and RfPB programmes between 1 January 2005 and 31 December 2015 were included. In the absence of universally adopted definitions of surgical interventions and PFS, for the purposes of this review, pilot/feasibility work was defined as: any research that is undertaken before a main study and is explicitly intended to inform the design and/or conduct of a future main study*,* where main study is defined as a definitive study or RCT of an intervention(s). A surgical intervention was defined as: a diagnostic, therapeutic or adjunctive invasive intervention performed by a trained clinician, using hands, instruments and/or devices, and included operative, radiological and endoscopic procedures.

Internal pilot studies were excluded owing to growing opinion among trial methodologists that internal pilots do not meet the true definition of pilot studies^31^. This is because internal pilots are very distinct from external pilots in their methodology, being designed and funded as a part of a main trial with all data generated from this first phase contributing to the final analysis. Internal pilots are, therefore, most often used when no substantive changes to key components of the trial, such as the intervention or outcomes, are anticipated. In addition, study protocols of RCTs with an internal pilot phase usually include only limited detail regarding the internal pilot phase itself, such as a list of proposed progression criteria. It was therefore considered that a review of trial protocols with an integrated internal pilot phase would be of limited value for the purpose of this work. Also excluded were funded systematic reviews that did not state any intention to inform a future definitive study, and studies that focused on the evaluation of co‐interventions to surgery, for example the administration of anaesthetic drugs, and postoperative rehabilitation or enhanced recovery programmes. This was because the primary focus of this work was to explore the specific difficulties surrounding studies of surgical interventions.

### Data extraction and analyses

Data were extracted using a standard database developed in Microsoft Excel® (Microsoft, Redmond, Washington, USA), including general study characteristics, available data sources in addition to the study protocol (published papers) and the surgical specialty of the study. The data extraction form is available in *Appendix S1* (supporting information). Details of the study design (randomized or non‐randomized; quantitative or qualitative) and conduct, including characteristics of the patient population, were extracted. A framework was developed for capturing the uncertainties and challenges regarding the viability of a future main trial, informed by expert knowledge and previous methodological work regarding the design, definitions and reporting of PFS^11^, ^12^, ^13^, ^14^, ^15^, ^16^, ^17^, ^18^, ^32^, ^33^, ^34^, published MRC guidelines^19^ and the IDEAL framework^20^, ^21^. All possible reasons identified for undertaking PFS were grouped into five key domains: main trial design; logistics; recruitment; intervention; and outcomes. The domains were constructed and ordered according to how extraction progressed, with cross‐checking between authors. Special consideration was given to uncertainties and challenges considered more specific and/or relevant to surgical trials. Results were analysed in Microsoft Excel®. Descriptive statistics are reported with comparison between the HTA and RfPB, and randomized and non‐randomized cohorts where relevant.

## Results

### Screening

**Figure 1:**
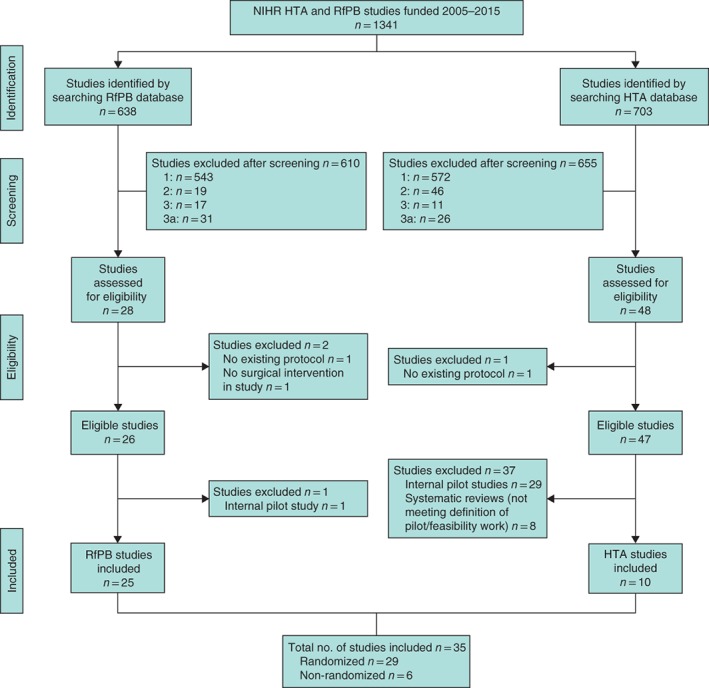
Flow diagram showing selection of articles for reviewReasons for exclusion: 1, not a surgical intervention; 2, surgical intervention, not pilot/feasibility work; 3, surgical intervention is a co‐intervention; 3a, surgical intervention is a co‐intervention and not pilot/feasibility work. NIHR, National Institute for Health Research; HTA, Health Technology Assessment; RfPB, Research for Patient Benefit.

**Figure 2:**
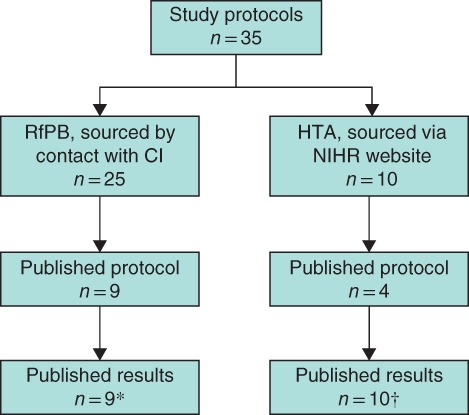
Data sources available for the pilot and feasibility studies included in the review*Published in peer‐reviewed journal. †Health Technology Assessment (HTA) report +/– other paper(s) in peer‐reviewed journal. RfPB, Research for Patient Benefit; CI, chief investigator; NIHR, National Institute for Health Research.

A total of 1341 funded studies were identified (703 HTA studies, 52·4 per cent), and 35 eligible studies (25 RfPB, 10 HTA) were included in the final analysis (*Fig*. 1). Additional data sources were available for 25 of the 35 studies; these included a published protocol paper for 13 of 35 studies and a paper reporting the PFS findings for 19 (*Fig*. 2). Publication rates of the study protocols were similar between funders (HTA 4 of 10, RfPB 9 of 25), although the results of HTA studies were published more often than those of RfPB studies (HTA 10 of 10, RfPB 9 of 25).

### Study characteristics

Some 29 of 35 studies were randomized and six were non‐randomized (*Fig*. 1), including qualitative work, national audit and cohort studies (*Table* 1). Randomized studies were more often multicentre and conducted by larger trial teams than non‐randomized studies. Over half of the randomized studies (15 of 29) also included other types of pretrial work.

**Table 1:** Design characteristics of the 35 pilot/feasibility studies in the review

	Randomized studies (*n* = 29)			Non‐randomized studies (*n* = 6)		
	RfPB (*n* = 22)	HTA (*n* = 7)	Total (*n* = 29)	HTA (*n* = 3)	RfPB (*n* = 3)	Total (*n* = 6)
**Surgical specialty of study**						
Gastrointestinal	8	1	9	2	0	2
Urology	3	2	5	0	0	0
Cardiothoracic	3	0	3	0	0	0
Orthopaedic	2	2	4	0	0	0
Obstetrics/gynaecology	2	0	2	0	1	1
Maxillofacial/ENT	2	1	3	0	1	1
Plastics	1	0	1	0	0	0
Paediatrics	1	1	2	0	1	1
Breast	0	0	0	1	0	1
**No. of centres** *	3 (1–23)	4 (2–10)	3 (1–23)	1 (1)	2 (1–65)	1 (1–65)
1	7	0	7	3	1	4
2–20	14	7	21	0	1	1
> 20	1	0	1	0	1	1
**Proposed no. of participants in study** *	50 (30–200)	70 (60–144)	60 (30–200)	n.a.	n.a.	n.a.
**Patient characteristics**						
Age						
Adults	19	5	24	2	2	4
Children	2	1	3	1	0	1
Both	1	1	2	0	1	1
Sex						
Male	1	1	2	0	0	0
Female	3	0	3	1	1	2
Both	18	6	24	2	2	4
Country						
UK	20	7	27	2	3	5
Europe	2	0	2	0	0	0
Worldwide	0	0	0	1	0	1
**No. of personnel in trial team** *	6·5 (4–19)	9·5 (0–24)	9 (0–24)	1 (0–12)	7 (2–25)	5·5 (0–25)
**Non‐randomized pretrial work**	9	6†	15†	3	3	6‡
Qualitative interviews	8	6	14	2	2	4
Participant/researcher survey	1	1	2	2	3	5
Economic modelling	0	1	1	0	0	0
Systematic review	0	0	0	1	0	1
National audit	0	0	0	0	1	1
Cohort study	0	0	0	0	1	1

* Values are median (range).

† Some studies planned more than one type of non‐randomized work;

‡ most studies planned more than one type of non‐randomized work. RfPB, Research for Patient Benefit; HTA, Health Technology Assessment; ENT, ear, nose and throat; n.a., not applicable.

### Reasons for conducting pilot/feasibility studies

Reasons for performing PFS are summarized in *Table* 2. Addressing uncertainties around trial recruitment was cited as the most common reason (32 of 35 studies; RfPB 23 of 25, HTA 9 of 10), followed by the overarching aim to determine whether a main trial was possible or necessary (27 of 35 studies; RfPB 17 of 25, HTA 10 of 10). Logistical issues (such as trial paperwork, resources needed, running multicentre studies) were considered in two‐thirds of studies (23 of 35 studies; RfPB 16 of 25, HTA 7 of 10) and outcomes (for example selecting the primary outcome, determining important outcomes for patients) in less than half (15 of 35 studies; RfPB 11 of 25, HTA 4 of 10). Around half of the studies considered issues regarding the sample size for the main trial (for example assessing variability in outcomes) (19 of 35 studies; RfPB 17 of 25, HTA 2 of 10) and costs/funding for the main trial (16 of 35 studies; RfPB 14 of 25, HTA 2 of 10).

**Table 2:** Rationale as detailed in the protocol for the pilot and feasibility studies of surgical interventions included in the systematic analysis

		No. of studies stating each rationale in the study protocol			No. of studies stating examination of each area in the study protocol		
Area examined	Rationale	RfPB (*n* = 25) (3 NR)	HTA (*n* = 10) (3 NR)	Total (*n* = 35) (6 NR)	RfPB (*n* = 25)	HTA (*n* = 10)	Total (*n* = 35)
**Main trial design**							
Main trial possible /necessary	To examine and test whether a main trial is possible	14 (2)	8 (1)	22 (3)			
	To assess whether main trial is needed	3 (0)	0 (2)	3 (2)			
	and/or produce a protocol				**17**	**10**	**27**
	To test whether the protocol can be adhered to and modify it as necessary	2 (0)	2 (0)	4 (0)			
Sample size	To estimate the variability in outcomes	15 (1)	2 (0)	17 (1)			
	to help determine a sample size for						
	the main trial				**17**	**2**	**19**
	To determine a sample size for the main trial	3 (0)	0 (0)	3 (0)			
Costs/funding	To assess/gather information on costs of performing the trial (direct and indirect)	2 (0)	0 (0)	2 (0)			
	To perform/prepare for a	13 (1)	2 (0)	15 (1)			
	cost‐effectiveness analysis of the						
	intervention(s)				**14**	**2**	**16**
	To provide information/evidence to funders	1 (0)	0 (0)	1 (0)			
Safety and effectiveness data	Preliminary data on safety to inform a main trial	2 (0)	0 (0)	2 (0)			
	Information on adverse events	4 (0)	0 (0)	4 (0)			
	Planned formal hypothesis testing of	3 (1)	0 (0)	3 (1)			
	safety outcomes*				**11**	**0**	**11**
	Preliminary data on effectiveness to inform a main trial	0 (0)	0 (0)	0 (0)			
	Planned formal hypothesis testing of effectiveness outcomes*	7 (1)	0 (0)	7 (1)			
**Logistics**	To test the logistics of multicentre studies	5 (0)	1 (0)	6 (0)			
	To develop a research network as a resource for a future main trial	1 (1)	0 (0)	1 (1)			
	To develop/test patient information content/forms/methods of delivery	1 (0)	3 (0)	4 (0)			
	To develop/test data collection forms/methods	13 (1)	6 (1)	19 (2)			
	To develop/test questionnaires/surveys	5 (0)	1 (0)	6 (0)			
	To test response rates to	0 (0)	0 (0)	0 (0)			
	questionnaires/surveys				**16**	**7**	**23**
	To prepare/plan/assess monitoring procedures	0 (0)	1 (0)	1 (0)			
	To determine what resources are needed for a main trial (e.g. funding/staff)	3 (0)	0 (0)	3 (0)			
	To assess the logistics of delivering an intervention as part of a trial in the NHS	1 (0)	0 (0)	1 (0)			
	To test (novel) methods of blinding	1 (0)	1 (0)	2 (0)			
	To assess proposed data analysis techniques	1 (0)	1 (0)	2 (0)			
	To learn about the day‐to‐day running of a trial	1 (0)	0 (0)	1 (0)			
**Recruitment**	To test/modify inclusion/exclusion/eligibility criteria	2 (0)	0 (1)	2 (1)			
	To estimate the expected prevalence or rate of incident cases in the population	1 (1)	1 (0)	2 (1)			
	To estimate the number to be screened and proportions of eligible patients	9 (0)	3 (0)	12 (0)			
	To assess numbers/rates of recruitment	17 (0)	5 (0)	22 (0)			
	and consent				**23**	**9**	**32**
	To test the randomization procedure	5 (0)	3 (0)	8 (0)			
	To test the acceptability of randomization/trial design	12 (1)	5 (2)	17 (3)			
	To determine the acceptability of the intervention to clinicians and patients	12 (1)	4 (2)	16 (3)			
	To assess rates of retention in the study	11 (0)	2 (0)	13 (0)			
**Intervention**	To assess and monitor the development of an intervention and/or its stability	2 (1)	1 (0)	3 (1)			
	To develop and test the implementation and delivery of the intervention	1 (0)	3 (0)	4 (0)			
	To train staff in delivery and assessment procedures	1 (0)	0 (0)	1 (0)			
	To monitor the surgical learning curve	2 (1)	0 (0)	2 (1)	**6**	**4**	**10**
	To test rates of crossover	0 (0)	1 (0)	1 (0)			
	To examine reasons for non‐adherence/crossover for the main trial	2 (0)	0 (0)	2 (0)			
	To develop pathways and protocols for co‐interventions	0 (0)	0 (0)	0 (0)			
**Outcome**	To select the most appropriate primary outcome measure	9 (1)	0 (0)	9 (1)			
	To develop and test a new outcome	0 (0)	0 (0)	0 (0)			
	measure				**11**	**4**	**15**
	To determine appropriate/important/suitability of outcome measures for patients/clinicians	3 (0)	4 (2)	7 (2)			

Values in parentheses are number of non‐randomized (NR) studies.

* Formal hypothesis testing to demonstrate the safety and/or effectiveness of an intervention is generally not recommended for pilot and feasibility studies because of the underpowered sample size (see discussion). RfPB, Research for Patient Benefit; HTA, Health Technology Assessment; NHS, National Health Service.

Eleven of the 35 studies (all RfPB‐funded) aimed to collect data regarding the safety or effectiveness of an intervention to inform the main trial and, of these, almost three‐quarters (8 of 11) specified plans for formal hypothesis testing by comparing the intervention(s) and/or control groups to test effectiveness and/or safety, which is not recommended for PFS.

One‐quarter (10 of 35 studies; RfPB 6 of 25, HTA 4 of 10) sought to explore uncertainties around the surgical intervention itself, such as intervention development, stability, delivery and the surgical learning curve. Of these ten studies, six were considering surgery *versus* no surgery, and four were considering a novel surgical technique *versus* an established method (*Table* 3). Of the ten studies specifically planning to evaluate a new surgical technique, only four aimed to address uncertainties surrounding the intervention. These uncertainties included: documenting the technical development of the new intervention to inform the development of a competency assessment tool for surgeon performance evaluation before participation in the main trial; considering the feasibility of training and implementing the new technique; determining the variation in the type of new procedure performed across the UK; and considering the impact of the learning curve on adverse outcomes to inform entry criteria for a main trial.

**Table 3:** Type of trial for studies that examined details of the surgical intervention and those that did not

	Studies examining the intervention (*n* = 10)	Studies not examining the intervention (*n* = 25)	Total (*n* = 35)
Surgery *versus* no surgery	6	9	15
New/novel surgical technique *versus* surgery	4	6	10
Non‐randomized pilot/feasibility work	0	5	5
Surgery *versus* surgery (both established techniques)	0	4	4
Surgery *versus* placebo and no surgery (2 arms)	0	1	1

## Discussion

This study demonstrated that the full potential of PFS to address the uncertainties and challenges specific to undertaking surgical trials is yet to be realized. The reasons most often cited by authors for performing PFS reflect the targeting of uncertainties generic to trials in general, such as recruitment, and considering whether a main trial is possible. Less than one‐third of surgical PFS explored challenges of specific relevance to designing and conducting trials in surgery, such as uncertainties around the stability or delivery of the surgical intervention itself. Notably, of the ten studies aiming to evaluate a novel surgical intervention, only four addressed uncertainties surrounding the procedure, such as development of the new intervention, implementation and delivery of the intervention, and the effect of the surgical learning curve. Of equal importance is the finding that the role of PFS in surgery is still often misunderstood, with nearly one‐quarter of studies planning to conduct formal hypothesis testing. Results of PFS in surgery are frequently under‐reported, with almost half not publishing the results to date, despite the majority having completed before 2018. These findings indicate that there is a need for guidance regarding the scope and optimal use of PFS to promote main trial success and prevent research waste.

There are several possible reasons for the findings observed in this study. Conceivably, there may be confusion among surgeons around the value of PFS. In addition, it is possible that the design of PFS in funding applications is skewed towards reasons perceived as important to funders, such as demonstrating adequate recruitment and the feasibility of completing a main trial to time and target. It is likely that most trialists would acknowledge that recruitment is paramount to study success. However, there may be a lack of awareness amongst applicants of the many other potential uncertainties that can compromise the success of a main trial, particularly those around the intervention.

Previous guidance^9^, ^10^, ^17^, ^18^, ^19^, ^35^, ^36^ regarding the optimal design and conduct of PFS has been theoretical or generic. The IDEAL framework, for example, describes a pathway for new surgical interventions from first in man (stage 1) to long‐term study (stage 4), with stage 2a (development) and 2b (exploration) studies considered to be PFS, focusing on addressing uncertainties before stage 3 assessment in a definitive RCT. The initial IDEAL publication^21^, however, was largely theoretical with little practical guidance about how PFS should be performed. Recently published updated IDEAL recommendations^22^ now provide some clarification regarding the role of PFS in surgery as a result of recognition that the original IDEAL guidance published in 2009^21^ had little impact on the design and conduct of surgical PFS^37^. The updated IDEAL framework^22^ suggests several feasibility issues to consider in stage 2a/2b studies, including estimating effect size, defining intervention quality and standards, evaluating learning curves, exploring subgroup differences, eliciting key stakeholder values and preferences, and analysis of adverse events. This list is far from exhaustive. The present analysis of protocols for NIHR‐funded surgical PFS indicates that there are important additional issues regarding the design and conduct of surgical trials that may usefully be explored in PFS.

The findings of PFS are often not widely disseminated^11^. This may reflect journal editors' lack of appreciation of the value of pilot work^11^, ^38^ and concerns about the quality or methodological rigour of pilot work in general^11^. Introduction of open‐access journals focused specifically on PFS such as *Pilot and Feasibility Studies* may address this^39^. Researchers themselves may also fail to prioritize the reporting and dissemination of findings from PFS, which may be fuelled further by the decision to pursue (or not) the main trial. Notably, however, all ten HTA‐funded studies identified in this review published their results. This is because studies funded by HTA are required to publish a report in the peer‐reviewed *Health Technology Assessment* journal.

The scope of PFS in general has historically often been narrow, focusing typically on issues relating to safety and efficacy^40^, ^41^ or recruitment^42^. A 2011 literature review^14^ of 50 pilot RCTs demonstrated that only 56 per cent of the studies addressed methodological issues in any depth. Another literature review^11^ of studies published between 2007 and 2008 found that up to 74 per cent of PFS performed and reported hypothesis testing for one or more variables (compared with 23 per cent (8 of 35) in the present study). There is now general acceptance that any suggestion of promise or significance should be reported with caution, given the underpowered sample size of most PFS^13^, ^15^, ^32^, ^43^, ^44^, and the present examination of protocols perhaps demonstrates a growing, if not complete, understanding of this issue.

This study demonstrated some key limitations of PFS in surgery, but focused solely on studies funded by the NIHR HTA and RfPB funding streams. Other NIHR funding streams were considered but excluded as they do not commonly fund surgical research. Of the 29 studies funded via the 2012 NIHR‐commissioned call for surgical research^45^, for example, only four met the inclusion criteria of the present study. These four studies were funded by the RfPB programme and were therefore all included in this work. Although the NIHR HTA and RfPB programmes are the major funders of studies of surgical interventions in the UK, it is accepted that there are charities, for example the British Heart Foundation and Arthritis UK, which may fund such work. However, this would have been logistically challenging given that very few funders make study protocols publicly available. Conversely, the NIHR‐funded protocols are likely to be of relatively high quality so may provide an overly positive perception of the quality of PFS in surgery.

Working collaboratively to design and perform pretrial work can deliver surgical PFS in a cost‐effective and timely manner, and can advance the development of definitive studies^46^. High‐quality PFS may also be resource‐ and cost‐effective, preventing waste by averting futile main trials, or providing information to improve the design and conduct of the main trial^47^. The findings from this study indicate, however, that PFS in surgery are not currently used to their full potential.

Work is under way to incorporate the present study findings into a wider methodological project to develop guidelines to support surgical researchers in undertaking PFS. These are likely to build on existing guidance from the MRC, the IDEAL group and broader methodological work, but focus specifically on surgical PFS. It will be important to explore if and how PFS influence main trial design and conduct. This will be achieved by following these NIHR‐funded PFS as they progress to main trials. The guidelines aim to provide researchers with clear and accessible information regarding how and when to undertake PFS, detail the key features to consider when designing and conducting PFS to inform a future main trial, and emphasize the importance of working collaboratively with trial methodologists to ensure that PFS address all uncertainties around future trial conduct accurately and wholly.

## Supporting information


**Appendix S1.** Data extraction form
