# Supplementary material for: Value of surgical pilot and feasibility study protocols
Source: Br J Surg. 2019 May 10;106(8):968–78. doi: 10.1002/bjs.11167 (PMC6618315; doi:10.1002/bjs.11167)
Supplement: Supplementary file 1 — Appendix S1. Data extraction form [file BJS-106-968-s001.doc]

**BJS11167**

**Value of surgical pilot and feasibility study protocols**

K. Fairhurst, J. M. Blazeby, S. Potter, C. Gamble, C. Rowlands and K. N. L. Avery

**Appendix S1 Data extraction form**

NB Detail is related to all proposed data points to be collected, including to enable follow up of studies as they complete and progress/not to a main trial

Allocated study code: NIHR Project number:

**Pilot and feasibility studies of surgical interventions:**

**A systematic review of NIHR funded studies.**

**DATA EXTRACTION FORM**

**(office use only)**

Please use all of the different data sources given to you to fill out this form. The form will in places ask for information from a specific source or guide you to where the information might be. Not all data sources are available for each study, but each study will have the minimum of a pilot/feasibility study protocol available. If data from a specific data source is needed (e.g. the pilot/feasibility results paper or study report) this will be stated.

**Background data**

**1** Date of data extraction (DD/MM/YYYY)

**2** Person extracting data If other please state

(1=KF, 2=KA, 3=SP, 4=JB, 5=CR, 6=other)

**3** Title of

**pilot/feasibility**

project

**4 Pilot/feasibility** project team personnel

| **Role in project**  1= CI, 2= Co-CI, 3= PI, 4 = Co-Investigator, 5= PPI representative, 6= Collaborator, 7= TSC, 8= TMC, 9=Sponsor representative, 10= Trial co-ordinator, 10= other (state) | **Specialty**  1= surgeon, 2=doctor, 3= Nurse/OPAM, 4=trialist/methodologist, 5= researcher, 6= qualitative researcher, 7= ethicist, 8= statistician, 9= Health economist, 10= R&D manager, 11 = other (state) |
| --- | --- |
|  |  |
|  |  |
|  |  |
|  |  |
|  |  |
|  |  |
|  |  |
|  |  |
|  |  |
|  |  |

**5** Please give any other information about the project team here

**6** Total number of people named in the protocol

**Section A: General characteristics of the study**

**A1a** What type of study do the authors label this as? Please tick all that apply.

(See guidance notes)

1= Randomised internal pilot 4= Other feasibility work

2= Randomised external pilot 5= Uncertain/not stated

3= Non-randomised pilot 6= Other

**A1b** If Other please state

**A1c** Verbatim description

**A2** Status of pilot/feasibility study? 1= Completed, 2= In progress, 3= Not completed/stopped, 4= Uncertain

**A3** Single centre Multicentre (X in appropriate box) If multicentre, state number of centres

**A4** Minimum number of proposed participants

**A5** Source of data **A5** Date of publication

(X in all available, see guidance notes) (Write date in box DD/MM/YYYY)

1

1

2

2

1= Pilot/feasibility study protocol

2= Pilot/feasibility grant application

3= Published pilot/feasibility protocol paper

3

3

4= Published pilot/feasibility study results paper

5= NIHR pilot/feasibility study report

4

4

6= Main trial protocol

7= Published main trial protocol paper

5

5

8= Published main trial results paper

6

6

7

7

8

8

**A6** How long did the pilot/feasibility study run for? (MM/YY)

**OR** uncertain/not stated

(please tick)

**A7** Surgical specialty of study 1=GI, 2=Urology, 3=cardiothoracic, 4=orthopaedic, 5=O&G, 6=MaxFax/ENT, 7=Breast,

8=Plastics, 9=Paeds)

**Section B: Rationale for the pilot/feasibility study**

**B1** Copy & paste *ad verbatim* any description of the rationale for the pilot/feasibility here (See guidance notes)

| **Area** | |  | **Intervention** | **B2a What were the reasons stated for performing the study in the protocol?**  (X each that apply – see guidance notes) | **B2b What areas did the study report on?** (X each that apply – see guidance notes) |
| --- | --- | --- | --- | --- | --- |
| Main Trial design | Main trial possible / necessary | 1 | To examine and test whether a main trial is possible |  |  |
|  |  | 2 | To assess whether main trial is needed and/or produce a protocol |  |  |
|  |  | 3 | To test whether the protocol can be adhered to and modify it as necessary |  |  |
|  | Sample size | 4 | To estimate the variability in outcomes to help determine a sample size for the main trial |  |  |
|  |  | 5 | To determine a sample size for the main trial |  |  |
|  | Costs / funding | 6 | To assess/gather information on costs of performing the trial (direct and indirect) |  |  |
|  |  | 7 | To perform/prepare for a cost effectiveness analysis of the intervention(s) |  |  |
|  |  | 8 | To provide information/evidence to funders |  |  |
|  | Hypothesis testing | 9 | To test the safety of an intervention |  |  |
|  |  | 10 | To test the effectiveness of an intervention |  |  |
| Logistics |  | 11 | To test the logistics of multicentre studies |  |  |
|  |  | 12 | To develop a research network as a resource for a future main trial |  |  |
|  |  | 13 | To develop/test patient information content/forms/methods of delivery |  |  |
|  |  | 14 | To develop/test data collection forms/methods |  |  |
|  |  | 15 | To develop/test questionnaires/surveys |  |  |
|  |  | 16 | To test response rates to questionnaires/surveys |  |  |
|  |  | 17 | To prepare/plan/assess monitoring procedures |  |  |
|  |  | 18 | To determine what resources are needed for a main trial (funding/staff) |  |  |
|  |  | 19 | To assess the logistics of delivering an intervention as part of a trial in the NHS |  |  |
|  |  | 20 | To test (novel) methods of blinding |  |  |
|  |  | 21 | To assess proposed data analysis techniques |  |  |
|  |  | 22 | To lean about the day-to-day running of a trial |  |  |
| Recruitment |  | 23 | To test/modify inclusion/exclusion/eligibility criteria |  |  |
|  |  | 24 | To estimate the expected prevalence or rate of incidence cases in the population |  |  |
|  |  | 25 | To estimate the number to be screened and proportions of eligible patients |  |  |
|  |  | 26 | To assess numbers/rates of recruitment and consent |  |  |
|  |  | 27 | To test the randomisation procedure |  |  |
|  |  | 28 | To test the acceptability of randomisation/trial design |  |  |
|  |  | 29 | To determine the acceptability of the intervention to clinicians and patients |  |  |
|  |  | 30 | To assess rates of retention in the study |  |  |
| Intervention |  | 31 | To assess and monitor the development of an intervention and/or it’s stability |  |  |
|  |  | 32 | To develop and test the implementation and delivery of the intervention |  |  |
|  |  | 33 | To train staff in delivery and assessment procedures |  |  |
|  |  | 34 | To monitor the surgical learning curve |  |  |
|  |  | 35 | To test rates of crossover |  |  |
|  |  | 36 | To examine reasons for non-adherence/cross-over for the main trial |  |  |
|  |  | 37 | To develop pathways and protocols for co-interventions |  |  |
| Outcome |  | 38 | To select the most appropriate primary outcome measure |  |  |
|  |  | 39 | To develop and test a new outcome measure |  |  |
|  |  | 40 | To determine appropriate/important/suitability of outcome measures for patients/clinicians |  |  |
| Other | |  | Other (state) |  |  |
|  |  |  | Other (state) |  |  |

**B2**  Categorisation of the rationale for the pilot/feasibility study (See guidance notes

**B3** Data related to the rationale for pilot/feasibility study**:** For each item identified in B2, please fill in B3a-c

(See guidance notes)

| **B3a. Reason/Area of uncertainty identified as:** | | **B3b. Defined *a priori* in protocol?** | **B3c. Data collected/Measured by?** | | |
| --- | --- | --- | --- | --- | --- |
| **Number** | **Actual wording of reason identified** |  | **Data collected?** | **Methodology** | **Notes** |
|  |  | Yes  No | Yes  No | Quantitative  Qualitative  Mixed  Uncertain  Other  (please state)  Not measured  or reported  Info not  available |  |
|  |  | Yes  No | Yes  No | Quantitative  Qualitative  Mixed  Uncertain  Other  (please state)  Not measured  or Reported  Info not  available |  |

**B4**  What was the **main reason** for performing the study defined as (*ad verbatim* – generally overall aim)?

**B5 Overall,** did the reasons for conducting the **pilot/feasibility study** remain the same as per the pilot/feasibility study protocol when the study was done? *(Will need to refer to pilot/feasibility protocol AND published pilot study paper/NIHR report if available)*

1= Adhered to

2= Not adhered to

3= Adhered to and additional reasons added

4= Uncertain (information not available)

**B6 Please add any additional information here**

**Section C: Pilot/feasibility study design**

**C1a** Copy & paste *ad verbatim* description of study design given in pilot/feasibility study protocol here

**C1b** Type of pilot/feasibility study 1= Randomised internal pilot

(See guidance notes) 2= Randomised external pilot

3= Non-randomised pilot

4= Other feasibility work

**C1c** If other specify

(See guidance notes)

1= Yes

**C1d** If reported, is the pilot/feasibility actually a small RCT? 2= No

(See guidance notes) 3= Uncertain/information not available

**C2**  Patient population of the **pilot/feasibility study** (X all that apply)

Adults (≥ 18) Male UK

Children (<18 yrs) Female Europe

Both Worldwide

Countries (List all)

**C3** Intervention of the **pilot/feasibility study** (If applicable, X all that apply, See guidance notes)

**Total number of study groups**

(Including intervention groups)

**What is the intervention group?**

Diagnostic

Operative Adjunctive

Therapeutic

Diagnostic

Surgical Radiological

Adjunctive

Therapeutic

Diagnostic

Endoscopic

Adjunctive

Therapeutic

**Name of interventions/Notes**

**C4** Comparators of the **pilot/feasibility study**

(If applicable, enter **number** of each type of comparator group in the appropriate boxes, see guidance notes)

**What are the comparator study group(s)?**

Best medical therapy Expectant management Pharmacological Usual/standard care

Diagnostic

Operative Adjunctive

Therapeutic

Diagnostic

Surgical Radiological

Adjunctive

Therapeutic

Diagnostic

Endoscopic

Adjunctive

Therapeutic

Other If other please state

No comparator interventions

**Name of comparators/Notes**

**C5** Outcomes of the **pilot/feasibility study** - If the protocol states primary/secondary/feasibility outcomes please copy and paste text *ad verbatim* here (See guidance notes)

**Primary:**

**Secondary:**

**Feasibility:**

**C6** Copy & paste *ad verbatim* description of data analysis proposed/used here

1= Yes

**C7a** Was a sample size calculation performed for the **pilot/feasibility study?**  2= No

3= Uncertain/info not available

**C7b** If performed, what reasons for doing so are given?

**C7c** If performed, what assumptions were made in order to make the calculation?

**C8a** Was an economic analysis done for the **pilot/feasibility study?** 1= Yes

2= No

3= Uncertain/information not available

**C8b** If yes, what were the stated end points for the pilot/feasibility economic analysis?

**C8c** If yes, what end points did the pilot/feasibility study actually measure?

**Section D: Outcome of pilot/feasibility study & progress of main trial**

*(Use all available data sources)*

1= Yes

**D1a** Does the report state that a definitive **main trial** is planned? 2= No

3= Uncertain/Information not available

**D1b** If yes, please categorise 1= Planned, 2= Not planned, 3= Uncertain/Information not available

1= Yes

**D2a** Does the report state that a definitive **main trial** has been funded? 2= No

3= Uncertain/Information not available

**D2b** If yes, please categorise 1= Funded, 2= Not funded, 3= Uncertain

**D3** If a **main trial** is planned/funded what is the status of the main trial?

1= Completed, 2= In progress, 3= Not completed/stopped, 4= Uncertain, 5= Not applicable

**D4** If a **main trial** is planed/funded/in progress, who are the project team personnel?

| **Role in project**  1= CI, 2= Co-CI, 3= PI, 4 = Co-Investigator, 5= PPI representative, 6= Collaborator, 7= TSC, 8= TMC, 9=Sponsor representative, 10= Trial co-ordinator, 10= other (state) | **Specialty**  1= surgeon, 2=doctor, 3= Nurse/OPAM, 4=trialist/methodologist, 5= researcher, 6= qualitative researcher, 7= ethicist, 8= statistician, 9= Health economist, 10= R&D manager, 11 = other (state) |
| --- | --- |
|  |  |
|  |  |
|  |  |
|  |  |
|  |  |
|  |  |
|  |  |
|  |  |
|  |  |
|  |  |

**D4b** Please give any other information about the project team here

**D5a** Are there any stated decision/progression criteria regarding progress to a **main trial**?

(See guidance notes) 1= Yes

2= No

3=Uncertain/information not available

**D5b** If yes, copy and paste text *ad verbatim* here

**D5c** Were these decision/progression criteria met? 1= Yes, 2= No, 3= Uncertain/info not available, 4= Not applicable

**D5d** If no, why were these criteria not met? Copy and paste text *ad verbatim* here

**D6a** Were remaining uncertainties about the **main trial** reported? 1= Yes, 2= No, 3= Uncertain/info not available

**D6b** If yes, please copy and paste *ad verbatim* any text regarding remaining uncertainties about the feasibility/viability of the main trial here

**D7** If main trial **completed/in progress**, how was it improved?

**D8** If the main trial is **not completed/stopped**, why did this happen?
